# THE EARLY BIRD GETS THE WORM, NOT ANXIETY OR DEPRESSION: HOW INSOMNIA SYMPTOMS PREDICT ANXIETY AND DEPRESSION

**DOI:** 10.1093/geroni/igz038.174

**Published:** 2019-11-08

**Authors:** Courtney J Bolstad, Anisha L Thomas, Michael R Nadorff

**Affiliations:** Mississippi State University, Mississippi State, Mississippi, United States

## Abstract

Symptoms of insomnia are associated with symptoms of depression and anxiety in older adults, yet less is known about the impact of specific forms of insomnia (i.e. onset, maintenance, and terminal insomnia). We explored how insomnia type predicted symptoms of anxiety and depression in older adults (n = 133; mean age 69, range 65-89). We hypothesized that onset and maintenance insomnia would have stronger relations to depression and anxiety than terminal insomnia. Regression analyses indicated that onset insomnia was the only significant predictor of anxiety symptoms, and maintenance was the only significant predictor of depressive symptoms. Thus, our findings suggest that despite overlap between depression and anxiety, insomnia may have different mechanisms of affecting each disorder. Implications for the treatment of anxiety and depressive symptoms by addressing insomnia problems will be discussed.

